# The Relation of Ametropia to Blepharitis Ciliaris

**Published:** 1878-04

**Authors:** F. C. Hotz


### Original Communications.

### THE RELATION OF AMETROPIA TO BLEPHARITIS CILIARIS.

By F. C. Hotz, M. D.

In a great many cases of blepharitis ciliaris* which present themselves for treatment, the disease has persisted for a period of several months, if not years. Perhaps the patient has never had any treatment for his lid trouble and thus its persistence is not to be wondered at. But often we are informed that the patient has been under the care of experienced oculists; that the lids improved and seemed to get well; but that sooner or later the inflammation recurred. Such patients of course, become rather discouraged by this experience and physicians who have occasion to observe their frequent relapses are misled into the belief that blepharitis is a very intractable disease. And yet it is far from being so. Its obstinacy is more apparent than real, and can, I think, be easily accounted for. We must only remember that the patient does not consider the disease dangerous because it does not directly impair his sight; that it is the unsightly appearance of the ulcerations and crusts upon his lids for which he seeks medical advice. By a few local applications these ulcers are so nearly healed that the edges of the lids do not become crusted over any longer. The defacement is removed, although the disease is not eradicated. But as the patient was concerned only about the disfigurement, he abandons treatment as soon as the cause of his anxiety is removed, and before a thorough cure can be accom-

* This term is now commonly used to designate the eczematous inflammation which shows itself along the edges of the eyelids and involves the follicles of the eyelashes (cilia). It is also called ophthalmia tarsi.

plished. The lids remain in a morbid state, and the most insignificant irritation suffices to rekindle the inflammation. This is not likely to occur if the treatment is properly continued until the lids do not show any infiltration or congestion or desquamation. But these are exceptional cases, and therefore a permanent cure of blepharitis is seldom recorded.

This explanation is not invalidated by the fact that many patients have used one or another remedy for blepharitis many months without any lasting benefit. I lay great stress upon this one point: that the surgeon himself must attend to the treatment, if it is to be efficient. For I have been convinced by numerous observations that to leave the local treatment of the lids to the patient or, if it is a child, to the parents, amounts to little or no treatment. Parents are too kind-hearted to insist upon thoroughly cleansing the crusted lids when at their first attempt the little darling begins to scream; and they are too timid to apply the ointment to the very edge of the lid. The remedy is either rubbed upon the crusts or smeared all over the cutaneous surface of the lid except its edge. And in either case it will be of just as much benefit as if it had not been used at all.

Under these circumstances it would be surprising, indeed, if a relapse of the disease were less frequent when the affected tissues have neither recovered their sound molecular structure nor resumed their normal functions. The untimely interruption, and the insufficient mode of the treatment, therefore, seem to me to fully explain, in the majority of cases of blepharitis, the unsatisfactory results so commonly obtained in private and hospital practice. And I do not think it is necessary to look beyond these very plain causes and attribute to the refraction of the eye any great influence upon the development of blepharitis ciliaris. This view of a direct relation between refraction and tarsal inflammation was first entertained by Dr. D. B. St. John Roosa, of New York. Before the International Ophthalmological Congress, held in New York, September, 1876, he undertook to show that "ametropia (anomalous refraction) seems to be the condition of most eyes affected with blepharitis ciliaris;" that "hypermetropia is the error of refraction most frequently associated with blepharitis ciliaris;" and that "when blepharitis is associated with errors of refraction, the cure of the edge of the lids is very much facilitated by, and sometimes depends upon, the correction of the ametropia." *

In support of these views Roosa presented a statistical report of 31 cases of blepharitis, of which 26, or $83\frac{9}{10}$ per cent., had refractive errors (13 hypermetropia, 5 myopia, 8 astigmatism) and only 5, or $16\frac{1}{10}$ per cent., had emmetropic eyes.

Dr. P. D. Keyser, of Philadelphia, confirmed Roosa's views by a report † of 31 cases, of which 25 had hypermetropic eyes, and 6 were astigmatic. But while Roosa is inclined to believe that ametropia only predisposes to the outbreak and favors the continuance or relapse of the lid trouble, Keyser goes a step further, and thinks that "accumulations of experience may show the fact of ametropia being in many, if not all cases, the direct cause of blepharitis." ‡

On the other hand, Dr. Ad. Alt, resident surgeon of the New York Ophthalmic and Aural Institute, says § :

" Since Dr. D. B. St. John Roosa, at the meeting of the international ophthalmological congress, held in New York in September, 1876, has advanced the idea that blepharitis ciliaris was, in about 83 per cent. of the cases, connected with ametropia, and holds ametropia a frequent cause of conjunctivitis and blepharitis, 48 patients have been especially examined with regard to that statement; 39 of them had emmetropia, 5 myopia, 3 hyperopia, 1 astigmatism — certainly no striking prevalence of ametropia."

My own observations during the past year extended over 18 cases, in private practice, of idiopathic || blepharitis, of which only 5 or 33 per cent. showed ametropia (4 hypermetropia, one myopia and one astigmatismus). The state of refraction was

---

* "The relations of Blepharitis ciliaris to Ametropia," in the report of the fifth International Ophthalmological Congress; also published in the *Amer. Journ. Med. Sciences*, January, 1877.

† "On some forms of inflammatory diseases of the eye being caused by defects of the refraction and accommodation," in the *Transactions of the Med. Soc. of the State of Pennsylvania*, 1877.

‡ *Phila. Med. Times*, 1877, p. 268.

§ "Clinical Report of 3,873 Eye-patients treated at the New York Ophthalmic and Aural Institute during the year 1876," in Archives of Ophthalm. and Otol., vol. vi., p. 180.

|| *I. e.*, not complicated by (or the sequelæ of) other inflammatory affections of the eye.

carefully ascertained by means of the ophthalmoscope, without, however, the previous use of atropia, which Keyser employed in all, and Roosa in the majority of cases. Though the accommodation becomes pretty well relaxed in the dark room, and although I had frequent opportunity for testing the reliability of my ophthalmoscopic measurement afterwards, when the eyes had been atropinized, and found it confirmed by the other tests—I admit that the influence of the ciliary muscle is not as completely suspended in the dark room as by the paralysing action of atropia; and I concede that, had I employed the atropia, some of my cases might have shown a slight degree of hypermetropia, and consequently increased the percentage of ametropia.

I am quite willing to make this concession; for I hope I shall be able to prove that the mere statistical fact that ametropia prevails among blepharitic patients, cannot be accepted as conclusive evidence of any etiological connection between the two affections. I think I can show that such deductions are as fallacious as the argument of *post hoc ergo propter hoc*, which in therapeutics has so often led to deception and disappointment.

In the first place, the high percentage of ametropia Dr. Roosa has found among his blepharitic cases, is nothing very extraordinary, since the examination of thousands of *atropinized* eyes has shown that ametropia is the rule and emmetropia the exception in the refractive state. F. Erisman,* of St. Petersburg, examining the refraction of the atropinized eyes of 4,358 pupils, aged from 6 to 20 years, found 30.2 per cent. myopia, 43.3 per cent. hypermetropia, 26 per cent. emmetropia and 0.5 per cent. amblyopia. In other words, 73.5 per cent. ametropia, against only 26 per cent. emmetropia; a result which comes pretty near the statistics of Dr. Roosa.

Those who believe in the causal relation between ametropia and blepharitis, refer, in advocating their views, to the well-known fact that the continued use of the eyes for near work (as reading, writing, sewing) often occasions a hyperæmia of the conjunctiva and the edges of the lids. This hyperæmia — they continue to reason—becomes chronic, produces an alteration of

* Graefe's Archiv. f. Ophthalmol., xvii., 1, p. 8.

the nutrition, and finally leads to an inflammation of the tissues involved. Says Keyser*:

"That ametropia of any kind or form causes in all acts of vision a strain more or less upon the eye, which creates a hyperæmic condition of the neighboring parts, is a well-known fact, as may be seen in many cases by red and congested conjunctiva and edges of the lids after use at close work or reading. In cases where the strain is so great as to create a continued hyperæmia of the edges of the lids, the extremely fine ducts and external openings of the small sebaceous glands that are to be found in the canals and follicles of the cilia, become closed by pressure from the swelling of the tissue and vessels surrounding them, and having no outlet for the natural secretions, which are now increased by the hyperæmic condition, a choked status results, and inflammation and suppuration take place, as may be noticed by the little pus beads that are found encircling the cilia and extending down the canal to the gland."

According to this view a great strain of ametropic eyes, during the act of near vision, is the primary condition which ultimately leads to the inflammation of the lids.

Now, in the first place, this accommodative strain does not take place in nearsighted eyes, unless they are armed with unsuitable glasses. We know that myopic persons can read or write almost day and night without feeling the least fatigue in their eyes, because their far point of vision is so near the eye that they have to make little or no effort at accommodating their focus for the short distance at which they wish to read or write. A person, for instance, with myopia 1-14 does not make the least accommodative effort while reading books printed in the usual types at the distance of twelve to sixteen inches, because if the accommodation of these eyes is completely relaxed, the farthest point of distinct vision is at 14 inches in front of these eyes; therefore if their eyes are perfectly at rest they can see distinctly at short distances to accommodate for which the normal (emmetropic) eye has to make a considerable effort. This exertion, if continued for hours, must necessarily exhaust

* Transactions of the Med. Soc. State of Pennsylvania, 1877, p. 536.

the muscular force of the emmetropic eye, and produce the sensation of fatigue; while the nearsighted eye does not get tired, and on this account is often erroneously taken for a "strong" eye.

In view of these facts it is impossible to recognize in myopia a source from which through a continued strain of accommodation and consequent hyperæmia of the lids, the tarsal inflammation could derive its origin.

It is a different thing with hypermetropic eyes. Hypermetropia, if not corrected by convex glasses, is the source of a continual strain upon the eyes. The accommodation of farsighted eyes is never relaxed except during sleep, because they must make more or less effort at accommodation even when they look at distant objects, in order to overcome the refractive error, and to gain a well-defined, focalized impression upon the retina. This same effort to correct the anomalous refraction, must be made during reading or writing, in addition to the amount of accommodative action which is required to change the focus from distant to near vision. It is evident, therefore, that hypermetropes use their eyes for near work under very unfavorable conditions; the abnormal strain upon the accommodative function must sooner or later exhaust the power of the ciliary muscle; the eyes become tired, heated, red and painful; the vision is obscured, and the patient is obliged to interrupt his work. People generally call such eyes "weak," and oculists use the technical term "asthenopia," to designate in one word all these symptoms due to the overwork of accommodation.

As long as hypermetropic eyes are not employed much for near work, and if there is but a slight degree of hypermetropia, the person will not be troubled by asthenopia, because the strain upon the accommodation is not sufficient to produce it. The presence or absence of asthenopia, therefore, gives us a pretty sure indication as to whether or not there is any great strain upon the eyes. And where this strain is not sufficient to give rise to asthenopic trouble, it is certainly not great enough to affect such distant parts as the eyelids, so intensely as to produce blepharitis.

Roosa found hypermetropia 1-48 to 1-30 in nine cases, and hypermetropia 1-24 to 1-8 in four cases; Keyser reported hypermetropia 1-48 to 1-30 in twenty cases, and hypermetropia 1-24 to 1-5 in 5 cases. This preponderance of the slight degrees of hypermetropia, which so seldom give occasion for asthenopic trouble, militates strongly against the idea that they should play an important rôle in the development of the lid trouble. If we actually admit that these slight degrees of hypermetropia can have so far-reaching an influence upon the lids, we shall find it very difficult to account for the fact that blepharitis is so seldom observed in the large number of hypermetropes who consult us on account of asthenopia, and whose eyes decidedly labor under a great, continued strain. I have always observed the rule to record any visible morbid condition of conjunctiva, lids, etc., in patients who consulted me on account of refractive anomalies. In examining such eyes I could not fail to detect even a slight degree of blepharitis. And yet, looking over my memoranda for the past years, I find only 4 cases of blepharitis among 267 cases of anomalous refraction. In all these cases the error of refraction was so marked that it impaired the function of the eyes, and in the most of the hypermetropic and astigmatic eyes produced more or less asthenopia ; and nevertheless the nutrition of the edges of the eyelids was not disturbed except in four cases. While on the other hand, where the edges of the lids were inflamed, the anomaly of refraction was as a rule so insignificant that under ordinary circumstances it did not give any inconvenience to the patient, and it required artificial means to establish its presence.

As especially conclusive evidence of the causal relation between hypermetropia and blepharitis, Keyser referred to one case, with these words: "Also in the case of June 5, 1876, the hypermetropia was, without doubt, the cause of the blepharitis; as the lids of only one eye were affected, and this was the hypermetropic one, while the other was normal and no defect of refraction could be found." *

But this case is not so positive a proof of any direct connection between the two affections as it seems at first sight. In fact, it

* Transactions of Medical Society of State of Pennsylvania, 1877, p. 536.

becomes a very doubtful argument as soon as we know that blepharitis may be limited to one eye also under altogether different circumstances, as the following case may illustrate :

On October 3, 1876, I was consulted by the parents of Felix M ——, aged 9 years, because they believed him to have become near-sighted. They remarked that when reading he would bring the book very close to the eye, and could read but a short time. Each eye showed V = $\frac{20}{20}$ which was deteriorated by even the weakest convex glasses. But he could read large print (Jaeger 7) only, and had to hold the book as near as six inches to the eye. The ophthalmoscope detected H. 1-16 ; otherwise the fundus was normal. He was given convex 24 and after having these glasses on 10 minutes, he could read smaller print (Jaeger 3) and at a greater distance than he could without glasses. The right upper lid was covered with crusts produced by extensive ulcerative blepharitis, while the other lids did not show any defect.

In this case the blepharitis was confined to one lid, but, unfortunately for Keyser's theory, both eyes showed the same degree of H. and the same amount of accommodative strain.

There is another fact which does not well agree with the view that the strain caused by ametropia is the principal cause of blepharitis. I refer to the fact that this disease is most frequently met with in children ; and if it is observed in older persons, they can generally date the first outbreak back to childhood. As a rule we can say the disease is developed at a time of life when the acts of vision do not demand any continued efforts of the eyes. My experience is that the greatest number of blepharitic patients is observed among children under five years of age. And I was surprised by finding that neither Roosa's nor Keyser's list contains a single patient under five years and that both tables include very few patients between five and ten years. Can they really not have observed any such cases, or did they omit them because the tests of vision cannot be employed in so young children ? As my experience is greatly at variance with their statements, I will give here a statistical résumé from my private and hospital records.

In private practice I observed during the four years, 1874 to

1878, 73 cases of blepharitis ciliaris. According to the age they can be classified as follows:

| | | | |
|---|---|---|---|
| Under 5 years | 27 cases, | or 37 | per cent. |
| Between 5 and 15 years | 22 " | " 30 | " |
| Between 15 and 20 years | 14 " | " 19 | " |
| Over 20 years | 10 " | " 14 | " |

The record book of the Illinois Charitable Eye and Ear Infirmary gives this information :

Total number of cases of blepharitis during 1876...34.

| | | |
|---|---|---|
| Under 5 years | 16 or 47 | per cent |
| Between 5 and 15 years | 11 or 32.3 | " |
| Between 15 and 20 | 2 or 6.0 | " |
| Over 20 | 5 or 14.7 | " |

Total number of cases of blepharitis during 1877...... 55.

| | | |
|---|---|---|
| Under 5 years | 19 or 34.5 | per cent. |
| Between 5 and 15 years | 21 or 38.2 | " |
| Between 15 and 20 years | 9 or 16.3 | " |
| Over 20 years | 6 or 11.0 | " |

The hospital and private records agree in proving the prevalence of blepharitis among children, and a careful inquiry into all the circumstances associated with the beginning of the disease would, in most cases, discover other and more potent causes than ametropia.

In a few instances it has been mentioned that the edges of the lids which had been treated unsuccessfully for years, got well without much treatment as soon as the refractive error was corrected by suitable glasses. These observations fully coincide with the experience so often gained in cases of conjunctivitis, that they prove to be rather obstinate to the usual topical treatment, but are speedily cured as soon as any anomaly of refraction has been corrected. In either case the rapid improvement in the condition of the lids must be attributed to the correction of the ametropia; there is no doubt of that. But this improvement takes place, not because by correcting the refraction we remove the original cause of the disease, but because we abolish thereby a condition which is apt to aggravate and prolongate an existing inflammation of the conjunctiva or lids. Just as the least amount of smoke in the air, which does not make any palpable impression upon a healthy conjunctiva, markedly irritates an already inflamed conjunctiva, so does the abnormal effort hypermetropic and astigmatic eyes have to exert in near vision, more strongly affect the eyes of the lids in the inflamed than in the healthy state. While any congestion produced in healthy eyelids will pass off without consequences, it will increase the inflammation and retard the recovery of inflamed lids. And this, I think, is the real relation of ametropia to blepharitis ciliaris; the affection of the lids may sometimes be rendered very obstinate through the adverse influence of hypermetropia or astigmatismus. We must look upon ametropia as a complication of blepharitis; and whenever the affection of the lids, especially in adults, does not readily yield to a proper treatment, we may find a certain degree of ametropia to be the cause of the obstinacy of the disease. If this is the case we should of course remove this, like any other complication, in order to put the lids in the most favorable condition for recovery.

